# Chagas disease mortality during the coronavirus disease 2019 pandemic: A Brazilian referral center experience

**DOI:** 10.1590/0037-8682-0562-2021

**Published:** 2022-02-25

**Authors:** Alejandro Marcel Hasslocher-Moreno, Roberto Magalhães Saraiva, Gilberto Marcelo Sperandio da Silva, Sergio Salles Xavier, Andréa Silvestre de Sousa, Andrea Rodrigues da Costa, Fernanda de Souza Nogueira Sardinha Mendes, Mauro Felippe Felix Mediano

**Affiliations:** 1 Fundação Oswaldo Cruz, Instituto Nacional de Infectologia Evandro Chagas, Rio de Janeiro, RJ, Brasil.; 2 Universidade Federal do Rio de Janeiro, Faculdade de Medicina, Rio de Janeiro, RJ, Brasil.

**Keywords:** Chagas disease, COVID-19, Mortality, Chronic heart disease, Comorbidity

## Abstract

**Background:**

We investigated the mortality rates of patients with Chagas disease (CD) during the coronavirus disease 2019 (COVID-19) pandemic and assessed the association between this mortality and CD clinical presentation and comorbidities.

**Methods:**

This was an observational retrospective study with clinical data retrieved from medical records.

**Results:**

Comorbidities were more prevalent among patients who died from COVID-19 than those who died from other causes. The proportion of patients according to CD clinical presentation was similar between the two groups.

**Conclusions:**

The prevalence of comorbidities seems to be related to a poorer prognosis in CD and COVID-19.

Severe acute respiratory syndrome coronavirus 2 (SARS-CoV-2) was first identified in Wuhan, China, in December 2019. Coronavirus disease 2019 (COVID-19) is an emerging infectious disease that causes a respiratory illness that is transmitted from person-to-person^1^. The lung is the organ most commonly affected by COVID-19, resulting in acute respiratory distress syndrome. In addition, SARS-CoV-2 may also damage other tissues, such as the heart. Myocardial injury is common in COVID-19, whereas clinical myocarditis is uncommon but may cause acute or prolonged heart failure and pericardial effusion^2^. The first case of SARS-CoV-2 infection in Brazil was identified at the end of February 2020. Since then, 1.3-3.2 million Brazilian patients with Chagas disease (CD) have been at risk of co-infection with this virus^3^.

CD remains a significant public health problem, with high morbidity and mortality in several Latin American countries, and is an important and neglected cause of death in Brazil. Between 2000 and 2019, 22,663,092 deaths were recorded in Brazil. CD was identified in 122,291 deaths (0.54%), and chronic Chagas heart disease (CHD) was the predominant clinical presentation^4^. CHD is the leading cause of death in patients with CD due to arrhythmias, heart failure, or stroke.

Chronic CHD pathogenesis is characterized by various disorders, such as inflammation, autoimmunity, fibrosis, dysautonomia, and microvascular changes. In turn, SARS-CoV-2 may cause myocardial injury, endothelial dysfunction, microvascular dysfunction, plaque instability, and myocardial infarction^5^. In theory, CD and SARS-CoV-2 coinfection can increase the pathogenic process, worsening CHD. However, information about the consequences of COVID-19 in patients with CD is scarce in the literature^6^. This study aimed to describe the mortality rates (both related to COVID-19 and other causes) of patients with CD at an infectious disease referral center in Brazil during the COVID-19 pandemic, and investigate its association with CD clinical presentation and comorbidities. Our hypothesis is that patients with CHD would have a higher mortality rate from COVID-19 when compared to patients with other clinical forms.

Patients with CD confirmed by two distinct serological techniques who attended the outpatient center of the Evandro Chagas National Institute of Infectious Diseases of the Oswaldo Cruz Foundation (INI-Fiocruz) from March 2020 to May 2021 were included in this observational retrospective study . The routine care of all patients included epidemiological history, directed anamnesis, physical examination focused on CD-related cardiovascular signs and symptoms, 12-lead electrocardiogram, and two-dimensional echocardiograms with Doppler. Clinical forms of chronic CD were classified according to the 2nd Brazilian consensus on Chagas Disease^3^. The cardiac form was classified into stages A, B1, B2, C, or D. Deaths were identified through an active search in the Extrajudicial Portal for consultations on births and deaths of the judiciary branch of the state of Rio de Janeiro. To facilitate data analysis, patients were grouped into indeterminate or cardiac form with normal echocardiogram, cardiac form with altered echocardiogram without heart failure, and cardiac form with heart failure. Comorbidities included arterial hypertension, diabetes mellitus, dyslipidemia, asthma, chronic obstructive pulmonary disease, and obesity. Descriptive statistics comprised the mean and standard deviation for continuous variables, and absolute and relative frequencies for categorical variables. Comparisons between groups according to the cause of death (COVID-19 vs. other causes) were performed using a two-sample t-test for continuous variables, and Fisher’s exact test for categorical variables. Dummy variables were created for each category of clinical form of CD (indeterminate; cardiac stages: A, B1, B2, C, D, and digestive) and number of comorbidities (none, one or two, and three or more). This strategy allowed a direct comparison between groups according to the cause of death (COVID-19 vs. other causes) for each of these categories. The Bonferroni correction for multiple comparisons (p-value multiplied by the number of tests) was performed to account for multiple comparisons between categories of clinical form of CD and number of comorbidities. The statistical significance was set at 5%. All analyses were performed using Stata version 13.0.

This study was approved by the INI-Fiocruz Research Ethics Committee (number CAAE: 37026320.2.0000.5262) on September 2, 2020 and was carried out in accordance with the 1964 Declaration of Helsinki and its later amendments. The need for informed consent was waived considering the retrospective nature of the study.

A total of 909 records were reviewed: 58.1% were women, with an average age of 64.5 +11.3 years. Thirty-five deaths were identified (3.9% all-cause mortality rate), 13 related to CD, 16 unrelated to CD (11 due to COVID-19), and 6 from unknown causes. Therefore, at least 31.4% of the total deaths were associated with COVID-19, representing a 1.2% COVID-19 mortality rate. The main characteristics of the deceased patients according to the cause of death are shown in Table 1. Of the 11 patients who died from COVID-19, most were women (81.8%). According to the clinical presentation of CD, there was no significant difference in the proportion of patients who died from COVID-19 and those who died from other causes (Table 1). All patients who died from COVID-19 had comorbidities. The prevalence of essential arterial hypertension, diabetes mellitus, and dyslipidemia was higher among those who died from COVID-19 than among those who died from other causes (Table 1), with an almost three-fold higher number of comorbidities (2.7 vs 1.0; p<0.001) in the first group. The percentage of patients with three or more comorbidities was also greater among those who died from COVID-19 than among those who died from other causes (Table 1).

**TABLE 1:** Characteristics of deceased patients according to cause of death (n=35)

Variable	Cause of death		*p-value*
	COVID-19	Other Causes	
	(n=11)	(n= 24)	
	Means (± standard deviation) or percentage (number of observations)		
Age (Years)	72.5 (+10.1)	69.9 (+10.7)	0.49
Women (%)	81.8 (9)	54.2 (13)	0.15
**CD classification - 2** ^nd^ **Brazilian Consensus (%)**			
Indeterminate Form	18.2 (2)	8.3 (2)	1.00*
CHD stage-A	36.4 (4)	20.8 (5)	1.00*
CHD stage-B1	0.0 (0)	16.7 (4)	1.00*
CHD stage-B2	9.1 (1)	4.2 (1)	1.00*
CHD stage-C	36.4 (4)	41.7 (10)	1.00*
CHD stage-D	0.0 (0)	8.3 (2)	1.00*
Presence of megaesophagus or megacolon	9.1 (1)	20.8 (5)	0.64
Essential arterial hypertension (%)	100.0 (11)	37.5 (9)	0.001
Diabetes mellitus (%)	54.5 (6)	16.7 (4)	0.04
Dyslipidemia (%)	81.8 (9)	37.5 (9)	0.03
Asthma / chronic obstructive pulmonary disease (%)	9.1 (1)	8.3 (2)	1.00
Obesity	27.3 (3)	4.2 (1)	0.08
Number of comorbidities (continuous)	2.7 (+0.9)	1.0 (+0.9)	<0.001
**Number of comorbidities (categories)**			
None	0.0 (0)	37.5 (9)	0.09*
One or two	36.4 (4)	58.3 (14)	0.87*
Three or more	63.6 (7)	4.2 (1)	<0.001*

*Corrected Bonferroni p-value for multiple comparisons.

The first study that reported cases of CD and SARS-CoV-2 coinfection included two patients (one indeterminate and one with CHD) who presented with rapid COVID-19 progression and died. The authors suggest that CD may be an important risk factor for developing severe COVID-19 and death, especially for patients with CHD, who may be more likely to develop poor outcomes due to their cardiac complications^7^. Despite the evidence that patients with a previous history of heart failure have a worse prognosis in the course of SARS-CoV-2 infection^8^ ^,^ ^9^, our results indicate that the percentage of COVID-19 mortality was similar across all clinical forms of CD in comparison to the percentage of deaths from other causes. A similar result was observed in a prospective multicenter cohort study including 37 hospitals in 17 cities from three Brazilian states that evaluated in-hospital outcomes from March to September 2020^10^. The authors concluded that CD and SARS-CoV-2 coinfection was not associated with worsening in-hospital outcomes. The possible mechanisms that explain similar COVID-19 and other causes of mortality among patients with CHD are still unclear. Notwithstanding, it may be considered that patients with CHD better adhered to preventive measures during the pandemic due to their fear of getting COVID-19 in the presence of a chronic heart condition, hence having less exposure to SARS-CoV-2, thereby decreasing their risk of COVID-19 death^11^. Conversely, some restrictive preventive measures, including quarantine and social isolation, may have negatively impacted the clinical management of CHD, increasing the risk of clinical decompensation and death by causes not related to COVID-19^12^. Therefore, the similar mortality rates for COVID-19 and other causes among patients with CHD observed in our study may have been circumstantial. More studies with larger sample sizes are needed to disentangle the prognostic influence of CHD on COVID-19 outcomes. 

The present study identified the presence of comorbidities as an important factor related to greater COVID-19 mortality, with a higher prevalence of arterial hypertension, diabetes mellitus, and dyslipidemia among those who died due to COVID-19. In addition, higher numbers of comorbidities were also associated with worse COVID-19 mortality rates because those who died from COVID-19 presenting almost three times more comorbidities than those who died from other causes. Therefore, this study shows that the greater the number of comorbidities, the worse the COVID-19 prognosis in patients with CD. These results are in line with previous studies, which show that, in addition to age, the presence of comorbidities increases the mortality risk in patients infected with SARS-CoV-2^13^ ^,^ ^14^. The exacerbated inflammatory cytokine response to COVID-19 infection is one of the mechanisms postulated for the worse prognosis related to the presence of comorbidities^15^. 

The retrospective study design should be recognized as a significant limitation that may introduce potential confounding factors for the relationship between the investigated factors (CD classification and comorbidities) and death. In addition, important information about the clinical course of COVID-19 (e.g., need for hospitalization in nursery or intensive care unit, day of onset of the disease at admission, lung and cardiac involvement at admission, days of hospitalization to death, thrombotic and other clinical events, necessity of mechanical ventilation, etc.) were not available, limiting data analysis. In addition, the small sample size precluded us from using more sophisticated statistical techniques such as logistic regression models to account for confounding factors. Therefore, these results should be interpreted with caution. Despite these limitations, to our knowledge, no previous study has evaluated the influence of clinical aspects of CD on COVID-19 prognosis, reinforcing the originality of the present findings.

To conclude, COVID-19 represented one-third of deaths in patients with CD at a referral center during the 14 months of the pandemic period. COVID-19 deaths were not apparently more significant in the presence of CHD, and therefore, do not seem to be a prognostic factor related to death in CD and SARS-CoV-2 coinfection. Conversely, the presence of multiple comorbidities seems to increase the risk of poor prognosis related to COVID-19.
